# A Case of Subphrenic Abscess with Interesting Features

**Published:** 1909-09-25

**Authors:** 


### A CASE OF SUBPHRENIC ABSCESS WITH INTERESTING FEATURES.

A subdiaphragmatic abscess is nearly always a symptom in the course of some other disease more or less remote; and, further than this, the infection is usually brought about by direct extension from a pre-existing focus in another viscus. Instances may be multiplied to prove this point. Reference to a few is, however, ample. An empyema, for instance, by extension downwards, or an appendicular or perirenal abscess by extension upwards, may equally infect the peritoneum on the under surface of the diaphragm; or, of course, an abscess may follow on the leaking of a gastric ulcer on the posterior wall of the stomach. But cases in which no such direct track can be found are comparatively rare, and it is mainly for this reason that the clinical history of the following case is interesting.

The patient, a man aged twenty-four, was first seen on June 30 of this year, complaining of pain in the right side of the chest and shortness of breath. A fortnight previously he had begun "to feel queer." He was treated for gastritis. A week before he had developed a dull aching pain in the left hip, which was worse if he stood up for any length of time. This had been regarded as rheumatic. At 5 p.m. on the day before he was first seen he had a rigor, and since then the shortness of breath and the pain on the right side referred to the region of the nipple had come on. He had had scarlet fever at the age of eleven, but no other illnesses. Syphilis was denied.

When seen the patient was flushed, and the alæ nasi were working, though he had not much apparent dyspnœa. The skin was dry and hot to the touch, and he had a dry cough and occasionally brought up some sputum, which was rather nummular in appearance. The clinical picture was, in fact, a typical one of lobar pneumonia, and the physical signs found in the right side of the thorax corroborated this hypothesis, the lower lobe of the right lung being consolidated. The temperature fell by lysis in five days, but never came down to normal, but a few days later (July 10) it rose again to 102° F., and remained about that level. The physical signs in the lung were unchanged; but as it was difficult to say whether the dulness found was due to solid lung or effusion, an exploring needle was put into the pleura, first in the sixth space in the posterior axillary line, and next in the seventh space in the line of the angle of the scapula. In neither case was fluid found. The temperature remained irregularly high, suggesting a focus of pus locked up somewhere. Further explorations were made from time to time, but without success until July 21, when a needle was inserted in the eighth space in the posterior axillary line and half an ounce of thick greenish pus, which did not smell offensive, was withdrawn. That afternoon a portion of the eighth rib was excised and the pleura opened. The lung appeared to be fully expanded, and no pus was found in the pleura. After this he continued much the same, with occasional fluctuations, but persistent pyrexia and dulness at the right base. He was again asperated on August 16 in the eighth space, above the wound of the operation, and more pus, similar to the last, was withdrawn. The wound was therefore opened up, and a further portion removed from the posterior end of the eighth rib. The cavity was explored, and on breaking down what appeared to be a firm adhesion at the base a large quantity of curdy, greenish pus was at once evacuated. A large empyema tube was put into the cavity and the wound was partially sewn up. The pus was examined bacteriologically; it contained pneumococci in great numbers, but these were apparently all dead, attempts to cultivate them failing altogether.

The patient was immensely improved by the operation; his temperature fell at once to normal, and the dulness at the right base diminished. He continued well for about a week until August 28, when his temperature suddenly rose during the night to 104.2° F. and he had a rigor. Next morning it was found that the character of the pus which came away through the tube was entirely changed. It was now thin and dark brown and had the peculiar odour associated with the bacillus coli. He also had hæmaturia.

The next day, August 29, he had another rigor. He now complained of pain in the abdomen, particularly on the right side, and on examination it was found that the abdomen did not move well and was rigid and tender in both flanks, more so in the right. A third operation was therefore performed, and the abdomen was opened by an incision parallel to and below the right twelfth rib. Immediately on opening the peritoneum which was thickened thin foul brown pus was discharged. No definite abscess cavity was found and there was diffuse peritonitis. Two tubes were inserted and the wound was partially closed. The patient vomited incessantly after the operation and died on the following day.

The autopsy revealed the following facts. The right lung was in a state of consolidation. The pleura was adherent to it all round. There was a hole in the diaphragm opposite the place where the rib had been resected. Underneath the right half of the diaphragm there was a large abscess cavity; and there was also diffuse peritonitis.

It must be presumed that, in this case, the subphrenic peritoneum was infected by the pneumococcus, without the intervention of an empyema, by means of the lymphatics. On the occasions on which pus was withdrawn by the exploring syringe, the needle must have gone through the diaphragm, which was pressed up by the abscess so that it lay almost parallel to the chest wall. Similarly when it was supposed at the second operation that a firm adhesion of the pleura was being broken down, it was in reality the diseased and softened diaphragm which was perforated with the finger. And, lastly, the final rise of temperature, coinciding with the change in the character of the pus, must have been caused by the bursting of the abscess into the general peritoneal cavity with consequent peritonitis, though it is still difficult to explain the almost immediate presence of the bacillus coli in the discharge.

